# Surface Alkylation of Cellulose Nanocrystals to Enhance Their Compatibility with Polylactide

**DOI:** 10.3390/polym12010178

**Published:** 2020-01-09

**Authors:** Joo Hyung Lee, Sang Ho Park, Seong Hun Kim

**Affiliations:** 1 Department of Organic and Nano Engineering, College of Engineering, Hanyang University, Seoul 04763, Korea; therauss@gmail.com; 2 LG Hausys R&D Center, Seoul 07796, Korea; babsang1002@gmail.com

**Keywords:** cellulose nanocrystals, surface modification, polylactide, nanocomposites

## Abstract

Effective surface alkylation of cellulose nanocrystals (CNCs) was developed using a nucleophilic substitution reaction with an alkyl bromide to convert hydrophilic groups on the CNCs into alkyl groups and the degree of substitution was quantitatively determined. The resultant alkylated CNCs exhibited improved dispersion in a nonpolar environment and increased hydrophobicity, compared with unmodified and acetylated CNCs. Polylactide (PLA) nanocomposites reinforced with unmodified and modified CNCs were prepared by a solution casting method and the effects of reinforcement on the thermal stability, mechanical properties, morphology, and barrier properties were investigated. In addition, modeling of the mechanical properties was evaluated to simulate the modulus of the PLA nanocomposites and results were compared with the experimental values. PLA nanocomposites reinforced with alkylated CNCs exhibited superior properties in terms of thermal stability, tensile strength, Young’s modulus, and barrier properties because of the uniform dispersion and strong interfacial adhesion between filler and matrix. This high performance and fully return-to-nature nanocomposite is expected to expand the utilization of CNCs from sustainable bioresources and the practical application of biodegradable plastics.

## 1. Introduction

Bio-based materials have attracted tremendous interest in both industrial and scientific fields because of their great potential for various high-value products with low impact on the environment. In this regard, cellulose nanocrystals (CNCs) are considered as an ideal nano reinforcement for biocomposites because of their outstanding mechanical properties, abundance, low density, inherent renewability, biodegradability, and biocompatibility [1,2,3,4]. The CNCs are generally isolated from natural cellulosic materials by acid hydrolysis, where their size and surface chemistry are dependent on the cellulose source and reaction conditions [5]. With a Young’s modulus as high as 145 GPa [6] and a large surface area of several hundred square meters per gram [7], the CNCs make it possible to enhance the physical properties of polymers remarkably at low filler loadings. However, due to their hydrophilic nature and abundance of hydroxyl groups, the homogeneous dispersion of CNCs is difficult to achieve in nonpolar solvents and most organic polymers, and thus CNCs often aggregate easily, resulting in poor compatibility between filler and hydrophobic polymer matrix [8,9]. The interface between filler and matrix is crucial for the microscale load transfer in composites and the moisture sorption induced by cellulose–water interaction leads to a reduction in the interfacial adhesion [10]. Abdelmouleh et al. reported the effect of moisture on the decrease of mechanical properties in grass pulp fibers/epoxy biocomposites [11].

As CNCs dispersible in a nonpolar environment have potential to realize useful CNC-polymer composites, extensive research into the addition of compatibilizers and/or surfactants and surface modification of CNCs has been carried out to prevent aggregation of CNCs and promote compatibility between the components. However, the compatibilizers which were possible to interact with CNCs or matrix would hinder the hydrogen bonding formation between reinforcing filler and matrix [12]. Furthermore, several studies have [8,13,14] reported the thermal degradation of matrix polymers by surfactants.

Various surface modification methods have been investigated; for example, acetylation by esterification between hydroxyl groups of CNCs and acetic anhydride in pyridine [10,15,16], transesterification so-called “grafting from” methodology for modifying CNCs with poly(lactide) (PLA) or poly(ε-caprolactone) by ring-opening polymerization [6,17], etherification [18,19], carbamation using isocyanates [20], succinication using succinic anhydride [21], carboxylation [22], and 2,2,6,6-tetramethylpiperidine-1-oxyl radical oxidation [23]. The reaction regions are limited to the accessible groups of the nanoscale entities [24]. For industrial applications, suitable methods for the surface modification of CNCs should be simple, economic, and efficient, without irreversible agglomeration in a nonpolar matrix. The surface modified CNCs have excellent dispersibility in a wide range of non-polar solvents such as chloroform, toluene, and etc. Furthermore, these surface modified CNCs also can be incorporated well into hydrophobic polymers.

Biomass-derived PLA is a very promising bio-material due to its outstanding mechanical properties, biodegradability, and commercial availability [25,26,27,28]. PLA can be utilized in the food packaging industry, where there is the trend that biodegradable plastics will replace petroleum-based plastics because of the lower environmental impact. However, several properties of PLA such as its gas permeability and thermal stability are somewhat insufficient. These properties should be further improved for specific applications [29]. Such improvements could be achieved by incorporating CNCs, homogeneously dispersed in a PLA matrix, for the reason of its chain immobilization effects as well as its excellent physical properties [30].

In this research, a facile method for surface modification of CNCs through a nucleophilic substitution reaction, which is faster and simpler than other functionalization methods [31], was performed; alkyl bromide was used in a one-step reaction. The effect of this surface modification method, in the PLA matrix, was compared to the acetylation method, which was one of the first methods used for the modification of cellulose fibers and which is still significant [32]. The chemical structure, degree of substitution, morphology, and hydrophobicity of surface modified CNCs were investigated. Finally, after the preparation of PLA/modified CNC composites, the transmittance, thermal stability, mechanical, and gas barrier properties were also investigated.

## 2. Experimental

### 2.1. Materials

Commercially available microcrystalline cellulose (MCC) with an average particle size of 20 µm was purchased from Sigma-Aldrich (St. Louis, MO, USA). PLA used as a polymer matrix was obtained from Cargill-Dow Inc. (4032D grade PLA, NatureWorks, Minnetonka, MN, USA); it had a molecular weight and density of 52,000 g/mol and 1.25 g/cm^3^, respectively. Sulfuric acid (98%) for the acid hydrolysis of MCC, chloroform as a solvent for PLA, dimethylformamide (DMF), and dimethylacetamide (DMAc) were purchased from Duksan Pure Chemicals Co. (Ansan, Korea). Acetic anhydride, pyridine, and 1-bromododecane were Sigma-Aldrich (St. Louis, MO, USA). All reagents were used without further purification.

### 2.2. Surface Modifications of CNCs

Details of the preparation of CNCs from MCC were described in our previous research [33]. In brief, the MCC was dispersed in deionized water and then sulfuric acid was added drop by drop, with vigorous stirring, until the appropriate concentration of 44% was reached. The resultant suspension was allowed to react for 120 min at 45 °C and then neutralized, by washing with deionized water repeatedly until the pH reached approximately 7. The product was filtered through a PTFE membrane.

An acetylation reaction was performed for the surface modification of CNCs. The reaction was carried out in a three-neck flask equipped with a condenser. First, powdered CNC (3 g) was added to DMF in an ice bath and ultrasonicated for 1 h to obtain a homogeneous dispersion. Second, pyridine (4.5 g) was added, followed by acetic anhydride (114 g), dropwise-acetylation then commenced. This process was carried out at 105 °C for 24 h, with vigorous stirring. Thereafter, the obtained suspension was washed repeatedly with a deionized water/methanol/acetone mixture (1/1/1 vol %) to eliminate by-products. The acetylated CNC (acetyl-CNC) gel was dried in vacuo at 60 °C for 48 h to afford the acetyl-CNC powder.

An alkylation reaction of CNCs was also carried out, by a nucleophilic substitution reaction using an alkyl bromide. CNC powder (2.1 g) was added to DMAc (60 mL) and ultrasonicated for 1 h. NaOH powder (6.17 g) was added and the reaction mixture stirred at room temperature under a nitrogen atmosphere for 1 h. The reagent 1-bromododecane (120.5 mL) was then added to the suspension drop by drop. The reaction was performed at 70 °C with continuous stirring for 24 h. After cooling, the product was washed repeatedly with a methanol/deionized water solution (8/2 vol %). Finally, the obtained samples were dried in vacuo at 60 °C for 24 h before the alkylated CNC (alkyl-CNC) was collected.

### 2.3. Determination of the Degree of Substitution by Titration

Substitution of the acetyl and alkyl groups on the surface of CNCs was determined by a titration method. Modified CNCs (50 mg) were dried in vacuo at 100 °C for 2 h, followed by immersion in a solution of methanol (15 mL)/deionized water (5 mL). The suspension was heated at 55 °C for 30 min in a lightly stoppered reaction flask. The next step was the addition of 1 N NaOH solution (20 mL) at 55 °C, over 15 min. Subsequently, the flask containing the suspension was tightly stoppered, and allowed to stand at 25 °C for 2 days.

The titration process, using phenolphthalein indicator, was conducted by adding 1 N HCl solution (20 mL) drop by drop until the red color appeared. The degree of substitution was calculated from the acetyl or alkyl content, based on HCl and NaOH consumptions. The degree of substitution of modified CNCs was plotted against the reaction time (Supplementary Figure S1).

Both acetyl-CNC and alkyl-CNC reacted for 15 h were used as reinforcements for PLA composites because of their similar degree of substitution values.

### 2.4. Fabrication of PLA/Modified CNC Composite Films

All the PLA composites were prepared by solution blending with the addition of acetyl-CNC or alkyl-CNC (0.5, 1, and 2 wt % in the polymer matrix). The modified CNC was incorporated into a chloroform solution of PLA, in an ice bath, under ultrasonic irradiation. PLA/modified CNC composite films were prepared on a glass plate by solution casting using a doctor blade. After slow evaporation of chloroform at room temperature for 24 h, the composite films were further dried under vacuum at 40 °C for 24 h. For comparison, neat PLA and PLA/CNC films were also prepared by the same procedure.

### 2.5. Characterization

Fourier transform infrared spectroscopy (FTIR; PerkinElmer Spectrum 2000, Waltham, MA, USA) equipped with a single reflection attenuated total reflectance system (Specac Ltd., London, UK) and solid state ^13^C CP-MAS NMR spectroscopy (Advance 400WB; Bruker, Billerica, MA, USA) were used to investigate the chemical structure of the CNC and modified CNC. The morphology of the CNC and modified CNC was investigated using a transmission electron microscope (Hitachi H-7600, Tokyo, Japan) at 80 kV and a field emission scanning electron microscope (JEOL JSM-6340F, Tokyo, Japan). The diameter and distribution of the nanofibers was measured by image analysis software (Image-Pro Plus, Media Cybernetics Inc., Rockville, MD, USA). Contact angle analysis with water and methylene iodide on the surface of CNC and modified CNC was measured using a contact angle goniometer (PXH300, SEO, Suwon, Korea). The images were recorded by a CCD camera after the droplet was deposited onto the specimen surface 1 min. An average of five measurements was made to evaluate the hydrophobicity of the surfaces. The volume of the individual droplet was 6 μL. The specimens were prepared by dropping 1 mL of CNC and modified CNC solutions on cover glass and drying the moisture repeatedly. The surface energies of CNC and modified CNC were calculated by the Owen–Wendt equation from the obtained contact angles. Thermogravimetric analysis (TGA) was carried out using a PerkinElmer Pyris 1 thermogravimetric analyzer, under a nitrogen atmosphere, at a heating rate of 10 °C/min. The mechanical properties of the PLA nanocomposites were measured using an instron tensile testing machine (UTM Instron 4465, Norwood, MA, USA) at room temperature. The gauge length and the crosshead speed were 50 mm and 1 mm/min, respectively. The reported tensile property values are average values from at least 10 specimens tested for each sample. UV-visible absorption spectra of the PLA nanocomposite films were investigated using a UV-visible spectrometer (S-4100, Scinco, Seoul, Korea) between 200 and 1000 nm. The gas barrier properties of PLA nanocomposite films were measured with a gas permeation analyzer (Mocon, OX-Tran 2/21 MD, Minneapolis, MN, USA).

## 3. Results and Discussion

### 3.1. Surface Modifications of CNCs

#### 3.1.1. Chemical Structures of CNC and Modified CNCs

**Figure 1:**
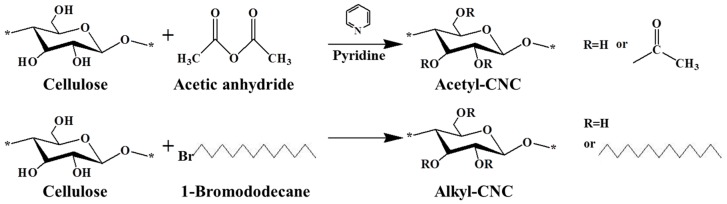
Synthetic procedure for surface modification of CNC.

To disperse CNCs homogeneously in a nonpolar environment, surface modifications were performed in order to convert hydroxyl groups on the CNC surface to acetyl or alkyl groups, as shown in Figure 1. Preparation of acetyl-CNC was carried out by the esterification reaction between hydroxyl groups and acetic anhydride. Preparation of alkyl-CNC was carried out by reaction with an alkyl bromide. First, the oxygen groups were deprotonated to O- ions under sodium hydroxide, followed by their reaction with 1-bromododecane in a one-step S_N_2 reaction, which is simpler and faster than other substitution reactions [34]. At reaction times <15 h, faster substitution was observed in the alkylation reaction of CNCs than the acetylation (Supplementary Figure S1). After 15 h reaction time, the value of the degree of substitution of alkyl-CNC became lower than that of acetyl-CNC, due to the steric hindrance effect of long alkyl chains on the CNC surfaces.

The chemical structures of modified CNCs were identified by FT-IR and ^13^C NMR analysis. The basic characteristics are summarized in Table 1. The peak located at 3350 cm^−1^ was assigned to O-H stretching of the CNCs. The acetylation of CNCs was confirmed by the appearance of a new peak around 1740 cm^−1^, associated with the carbonyl stretching vibration. The new peaks around 1370 and 1240 cm^−1^ were assigned to the methyl in-plane bending and C-O stretching vibration, respectively. Furthermore, the substituted alkyl groups in the alkyl-CNC were confirmed by detecting new peaks at 2853 and 2926 cm^−1^, corresponding to C-H stretching vibrations. The chemical structures of the surface modified CNCs were analyzed by ^13^C NMR. The typical NMR peaks of CNCs were assigned as follows: C_a_ (105 ppm), C_d_ (crystalline 89 ppm and amorphous 63 ppm), C_b_/C_c_/C_e_ (72–75 ppm), and C_f_ (crystalline 65 ppm and amorphous 63 ppm) [35]. After surface modification, the specific signals for the carbons of the acetyl group were detected at 172 ppm (C_α_) and 21 ppm (C_β_). In addition, the characteristic peaks of the carbons of the alkyl group emerged at 14–32 ppm, as summarized in Table 1. The chemical structure analysis indicated that most of the hydroxyl groups on the CNC surface were successfully converted to acetyl or alkyl groups by the surface modification.

#### 3.1.2. Morphology of Modified and Unmodified CNCs

**Figure 2:**
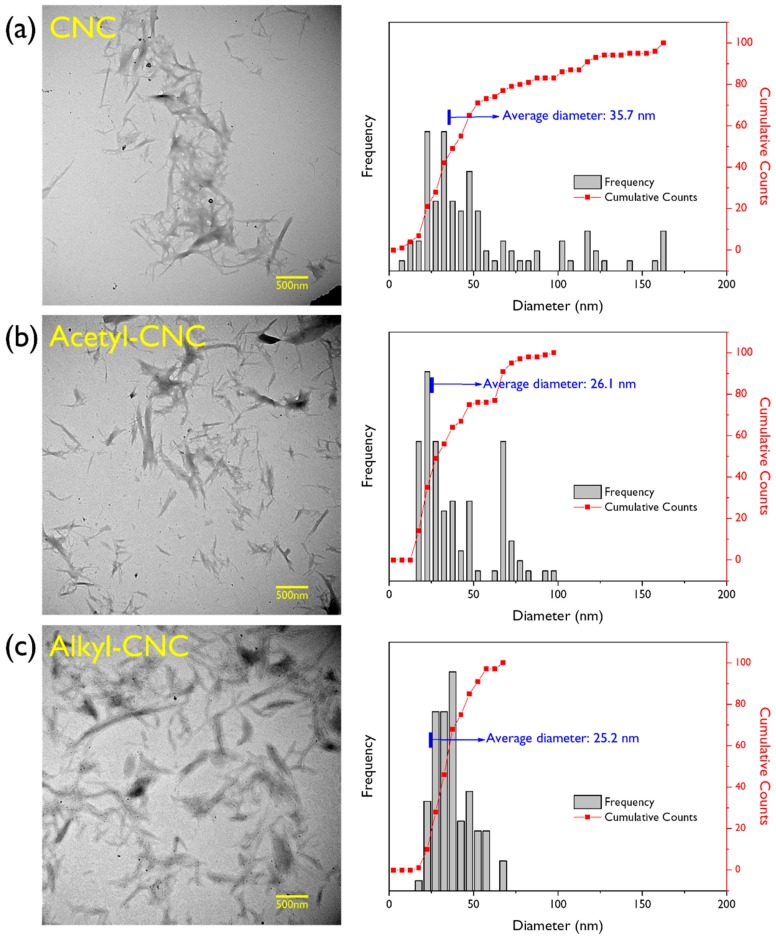
TEM images and diameter distributions of (**a**) CNC, (**b**) acetyl-CNC, and (**c**).

Analysis of both modified and unmodified CNCs was performed by transmission electron microscopy (TEM) to investigate morphological changes associated with the surface modification. As shown in Figure 2, the filler morphologies for modified and unmodified CNCs are similar in appearance. All three products (CNC, acetyl-CNC, alkyl-CNC) had a rod-like morphology with dimension diameters of approximately 20–30 nm. The average diameters of the CNCs, measured using image analysis software, decreased after surface modifications. Furthermore, a narrower distribution of CNC diameters was observed in alkyl-CNC than in acetyl-CNC, indicating a more uniform morphology without any change in intrinsic morphology and structure.

#### 3.1.3. Contact Angle Analysis and Surface Free Energy of CNCs and Modified CNCs

Contact angle analysis has been used to investigate the hydrophobicity, dispersity, and surface energy of materials [36]. As has been mentioned, CNCs have strong chain interaction as a result of hydrogen bonding, leading to a disturbance of dispersion in the hydrophobic polymer matrix. Surface modifications for improving the dispersion of CNCs was further confirmed by contact angle measurements. In general, there are three ways to measure the contact angle of a bulky solid materials in a powder state; (i) sessile drop, (ii) Wilhelmy plate and (iii) liquid penetration and Washburn capillary rise. Despite the accuracy of the Washburm method, static goniometric measurement was selected in this study due to lack of the CNCs and modified CNCs to make specimens. The average contact angles for water and methylene iodide on CNCs and the modified CNCs are summarized in Table 2. The value of the water contact angle of CNCs was <5°. Due to it being hydroxyl-group rich, it is more capable of hydrogen bonding with water. Acetylation treatment resulted in a dramatic increase in the water contact angle of CNCs, as reported in other literature [15,37]. Moreover, the alkyl-CNC had the highest water contact angle value, indicating that substituted alkyl chains have a greater effect on the surface polarity of CNC than the acetyl group.

The surface free energy of CNCs and the modified CNCs was determined by the Owens-Wendt approach, which divides the surface every into dispersive and polar contributions [38,39], according to the following equation:

(1) $$\gamma_{SL}=\gamma_{S}+\gamma_{L}-2\sqrt{\gamma_{S}^{d}\gamma_{L}^{d}}-2\sqrt{\gamma_{S}^{p}\gamma_{L}^{p}}$$

where the *γ*, *γ^d^*, and *γ^p^* are the total, dispersion, and polar surface energies, respectively and the subscripts L, S, and SL represented the liquid, the solid surface, and the solid-liquid interface, respectively. The solid-liquid interfacial energy can be replaced by the Young equation (*γ_S_* = *γ_L_cos θ* + *γ_SL_*), where *θ* denotes the contact angle of water and methylene iodide on the solid substrate) and the following equation is obtained.

(2) $$\gamma_{L}\left(1+cos\theta \right)=2\sqrt{\gamma_{S}^{d}\gamma_{L}^{d}}+2\sqrt{\gamma_{S}^{p}\gamma_{L}^{p}}$$

The liquid surface energies for water and methylene iodide were taken from the literature [40]. As summarized in Table 2, while the dispersion components of modified CNCs were not significantly changed, the polar components of modified CNCs were remarkably decreased by the introduction of hydrophobic groups to the CNC surface. This implies the good dispersion in the nonpolar solvents, which may be attributed to weakening of hydrogen bonding between CNC surfaces. As a result of the dispersion states of CNCs and modified CNCs in chloroform, after simply shaking, the suspensions of acetyl- and alkyl-CNC showed excellent dispersion and stability, as confirmed by the Tyndall effect (Supplementary Figure S2). Furthermore, the alkyl-CNC had a lower polar component value than the acetyl-CNC, suggesting that the facile modification of the CNC surface by alkylation is expected to result in a more significant improvement in the dispersion and interfacial adhesion in nonpolar PLA than otherwise, by acetylation (as a well-known modification of CNCs).

### 3.2. Properties of PLA Nanocomposites

#### 3.2.1. Fracture Morphology

**Figure 3:**
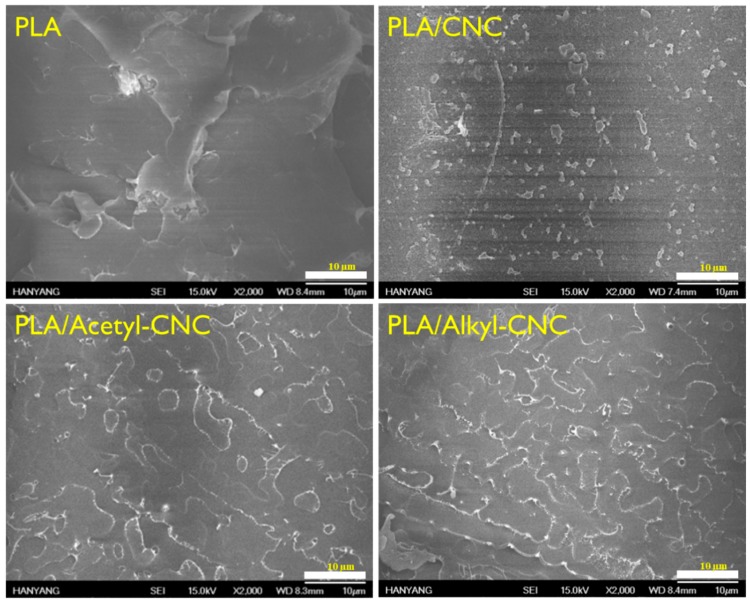
SEM images of PLA and PLA nanocomposites with 1 wt % CNC, acetyl-CNC, and alkyl-CNC.

The homogeneous dispersion of reinforcements in a polymer matrix is a crucial requirement for effective improvement of the matrix properties. Scanning electron microscopy (SEM) images of the fracture morphologies of the PLA and PLA nanocomposites are shown in Figure 3. Whereas the neat PLA presented a smooth fractured surface, CNCs in the PLA/CNC nanocomposite were not covered with immiscible PLA matrix and aggregated with uneven distribution. However, modified CNCs were homogeneously dispersed in the PLA matrix, indicating that the substituted acetyl and alkyl groups in modified CNC surfaces had good compatibility with PLA.

#### 3.2.2. Crystallinity

**Figure 4:**
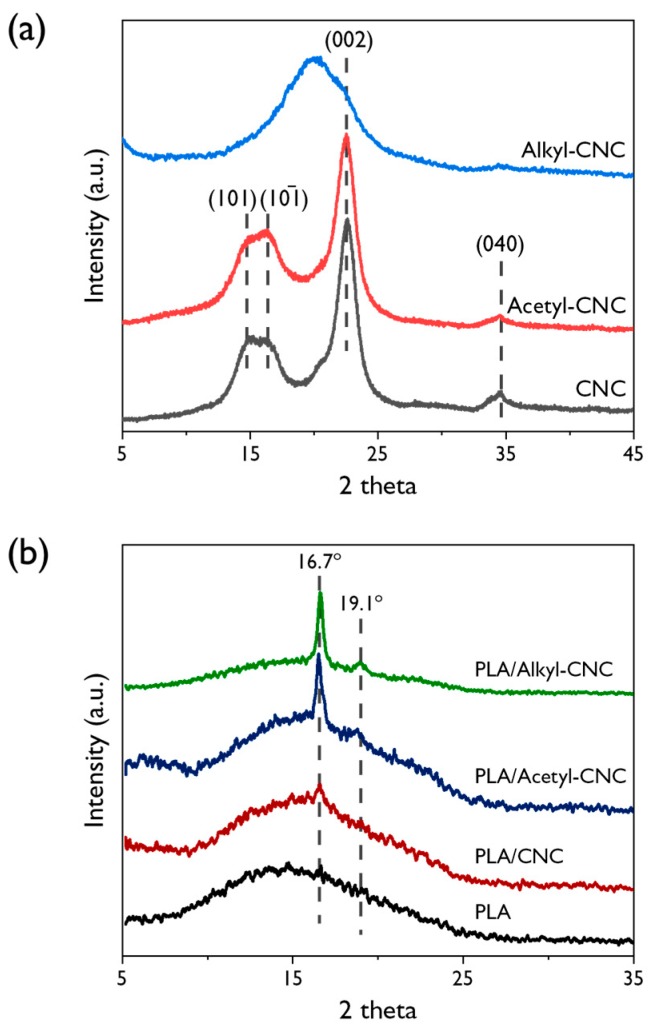
XRD patterns for the CNC, acetyl-CNC and alkyl-CNC (**a**), and XRD patterns for the PLA and PLA/CNC nanocomposites (**b**).

XRD patterns of the CNC, acetyl-CNC, and alkyl-CNC are shown in Figure 4a. The featured diffractions of the CNC and acetyl-CNC appear at 14.8°, 16.6°, 22.3° and 34.4°, corresponding to a typical pattern of cellulose I (101, 10$\bar{1}$, 002, and 040 lattices) [41]. This result indicated that the acetylation treatment had not affected the crystalline structure of CNC. The interplanar distance (*d*) is calculated from Bragg’s diffraction equation:

(3) 2dsinθ=nλ

where θ is Bragg’s diffraction angle, *n* = 1, and λ is the wavelength of the X-ray radiation (0.154 nm). The calculated interplanar distance for the Bragg angle of 22.3° in the CNC and acetyl-CNC is 0.20 nm. However, the alkyl-CNC has a relatively broad diffraction peak around 18.8° with a corresponding interplanar distance of 0.24 nm. This implies that the substituted alkyl chain increased the interplanar distance [42] with more amorphous structure.

As shown in Figure 4b, the neat PLA and the PLA/CNC films show the amorphous polymer patterns. However, the two slight peaks were observed around 16.7° and 19.1° corresponding to (200)/(110) and (203) reflections that were assigned to the ordered α phase of PLA crystal [43] in the PLA/acetyl-CNC and PLA/alkyl-CNC nanocomposites. This indicated that modified CNC resulted in the development of the crystalline structure of PLA by leading to chain packing of the crystal lattice. The typical peaks of cellulose I were not observed due to a low loading level of nanofillers.

#### 3.2.3. Thermal Stability

**Figure 5:**
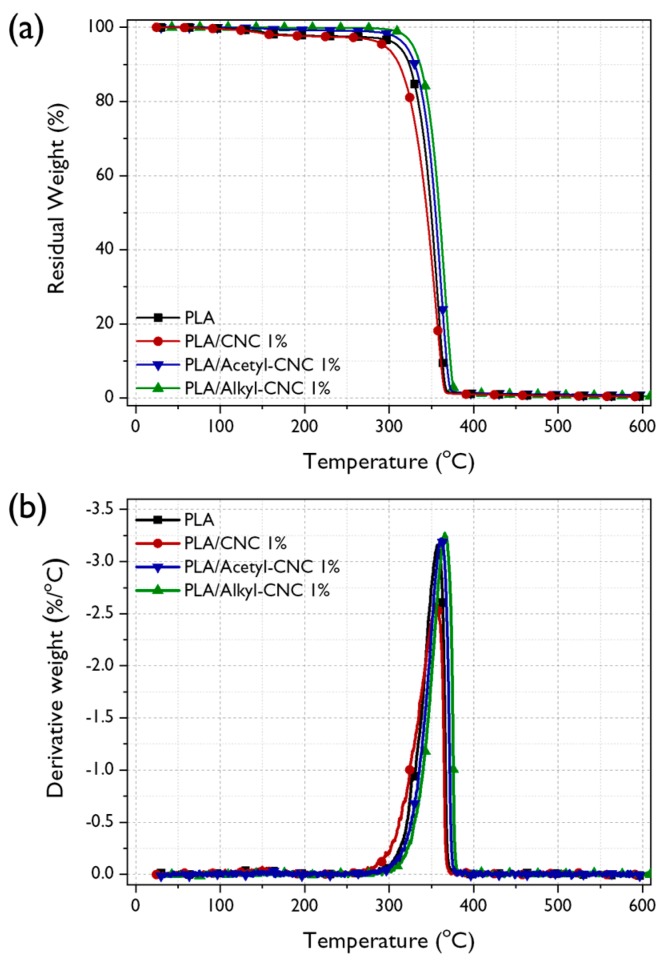
TGA thermograms (**a**) and DTG curves (**b**) of the neat PLA and the PLA nanocomposite.

The thermal stability of PLA is an important factor for its processing and applications because it influences the upper limit service temperature and the environmental conditions [44]. If PLA has low thermal stability, its application for food packaging materials, which involve melt processing technology, will be restricted. TGA to determine thermal stability of PLA and PLA nanocomposites was conducted. The TG and DTG curves are shown in Figure 5 and the results are also tabulated in Table 3. Lower thermal stability was observed in the PLA/CNC nanocomposite than neat PLA because the CNCs obtained from sulfuric acid hydrolysis might have released residual acid at the CNC surfaces, acting as catalyst for degradation of PLA above the melting temperature [45] and had poor adhesion with PLA. However, the incorporation of the acetyl-CNC and alkyl-CNC into the PLA matrix increased the thermal degradation temperatures as well as the temperature at maximum rate of weight loss (*T*_dm_) of the PLA nanocomposites. This enhancement was more remarkable in the PLA/alkyl-CNC nanocomposites. This result indicated that the thermal stability of PLA was improved by the presence of the acetyl-CNC and alkyl-CNC, which could act as effective physical barriers to prevent the thermal energy transportation. In addition, the PLA/alkyl-CNC nanocomposites exhibited further enhancement of thermal stability because the good interfacial adhesion between the alkyl-CNC and the PLA matrix might restrict the thermal motion of the PLA [46].

Kinetic analysis of thermal degradation was performed to clarify the effects of CNCs and modified CNCs on the thermal stability of the PLA nanocomposites. The activation energy for the thermal degradation (*E_a_*) of the PLA nanocomposites can be estimated from the TGA thermograms by the Horowitz–Metzger kinetic method [47], using the following equation:

(4) $$ln\left[ln{\left(1-\alpha \right)}^{-1}\right]=\frac{E_{a}\theta }{RT_{dm}^{2}}$$

where α is the weight loss and θ is the variable auxiliary temperature (*θ = T − T_dm_*). The *R* is the universal gas constant. The *Ea* for thermal degradation can be calculated from the slope of a linear fitting of *ln*[*ln*(*1 − α*)*^−1^*] versus *θ*, as shown in Supplementary Figure S3. The higher *Ea* value was obtained in the PLA/modified CNC nanocomposites, as compared with the neat PLA and PLA/CNC nanocomposite, implying that the PLA was more difficult to decompose because of addition of uniformly dispersed CNCs. Furthermore, this calculated *E_a_* values exhibited good reliance for describing the thermal degradation kinetics of the PLA nanocomposites because that the correlation coefficient (*r^2^*) values were >0.99.

#### 3.2.4. Mechanical Properties and Modeling

**Figure 6:**
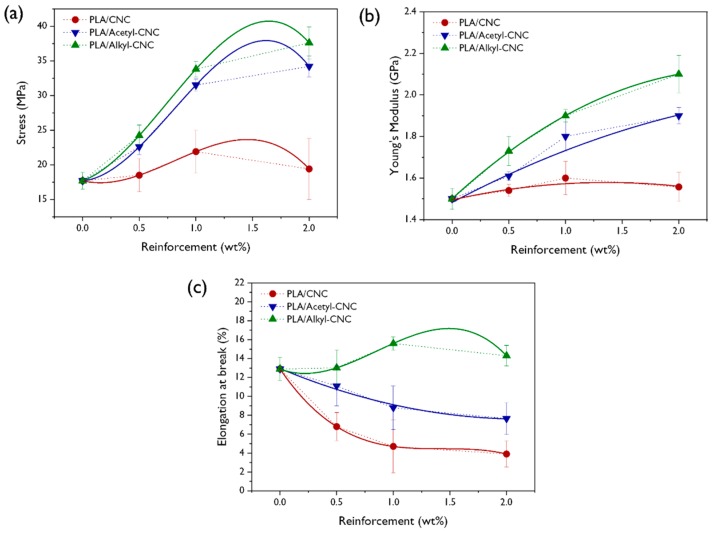
(**a**) Stress-strain curves, (**b**) young’s modulus, and (**c**) elongations at break of PLA/CNC, PLA/acetyl-CNC, and PLA/alkyl-CNC nanocomposites as a function of filler content.

The mechanical properties of neat PLA, PLA/CNC, and PLA/modified CNC nanocomposites are shown in Figure 6. It is well known that the PLA, when it is utilized for food packaging materials, should offer sufficient strength and stiffness, as well as resistance to handling damage [48]. The reinforcing effect of CNC on the mechanical properties of PLA was not remarkable, compared to that of acetyl-CNC and alkyl-CNC, due to the aggregation of CNCs and poor interfacial adhesion between PLA and CNCs. However, the tensile strength and Young’s modulus of PLA nanocomposites were increasingly improved with increasing acetyl- and alkyl-CNC contents because of the uniform dispersion and strong interfacial adhesion between fillers and PLA matrix. This positive behavior was more prominent in the PLA/alkyl-CNC nanocomposite, compared with the PLA/acetyl-CNC nanocomposite, implying the preferential interaction of the alkyl groups in the CNC surface with the amorphous PLA matrix than the acetyl groups in the CNC surface. Another noticeable feature was the slight increase of the elongation at break when the alkyl-CNC was incorporated into the PLA matrix. It is generally, well known that the addition of fillers results in decreased elongation at break in most composites, while the modulus of the composites is increases. Our result might be interpreted as follows: the uniformly dispersed alkyl-CNC restricts the stress-induced crystallization due to the possibility of alkyl-CNC disturbing the formation of the crystal lattice [49]. A similar observations has been reported, namely, the elongation at breaks of poly(ethylene terephthalate) nanocomposites is increased by the addition of functionalized graphene oxide [31]. Therefore, the alkyl-CNC effectively improved not only tensile strength and Young’s modulus, but also elongation at break, which would make the PLA matrix more ductile.

To characterize the reinforcing effect of modified CNCs on the mechanical properties of the PLA nanocomposites, the experimental results were compared with the values predicted from the theoretical models. Because the modified CNCs were considered to be randomly oriented in the PLA nanocomposites, the well-established Voight–Reuss [50] and Halpin–Kardos models [51] were used to predict the modulus of the PLA/modified CNC nanocomposites. The Voigt–Reuss model is one of the most common equations used for predicting the modulus; it involves the volume fraction of the filler (*V_f_*), in which the geometry of fillers is not considered, using only three independent variables. The modulus of composites (*E_c_*) predicted from the Voigt–Reuss model is calculated from the following equation:

(5) $$E_{c}=\frac{3}{8}\left[V_{f}E_{f}+\left(1-V_{f}\right)E_{m}\right]+\frac{5}{8}\left[\frac{E_{f}E_{m}}{E_{f}\left(1-V_{f}\right)+E_{m}V_{f}}\right]$$

where *E_m_* and *E_f_* are the moduli of the polymer matrix and filler, respectively. The Halpin–Kardos model is a semi-empirical equation for short-fiber oriented composites.

(6) $$E_{∥}=E_{m}\frac{1+\eta_{∥}\zeta V_{f}}{1-\eta_{∥}V_{f}},\;E_{⊥}=E_{m}\frac{1+2\eta_{⊥}V_{f}}{1-\eta_{⊥}V_{f}}$$

where

(7) $$\eta_{∥}=\frac{\frac{E_{f}}{E_{m}}-1}{\frac{E_{f}}{E_{m}}+\zeta },\;\eta_{⊥}=\frac{\frac{E_{f}}{E_{m}}-1}{\frac{E_{f}}{E_{m}}+2}$$

where *E*_‖_ and *E*_⊥_ are the expressions for the longitudinal and transverse Young’s modulus, respectively. *ζ* is a shape factor related to filler geometry and orientation. When the filler is of the short-fiber type, such as CNCs [52], the *ζ* is calculated by 2 *L*/*w* where *L* and *w* are the length and diameter of the filler, respectively. *E_c_* can be obtained by the following equation based on the laminate theory [53].

(8) $$E_{c}=0.184E_{∥}+0.816E_{⊥}$$

To fit equations to the experimental results, the weight fraction was transformed to the volume fraction, taking the densities of PLA (1.25 g/cm^3^) and CNCs (1.58 g/cm^3^) into consideration. The *E_f_* value of 167.5 GPa was used and the *E_m_* was determined from the tensile data [54].

**Figure 7:**
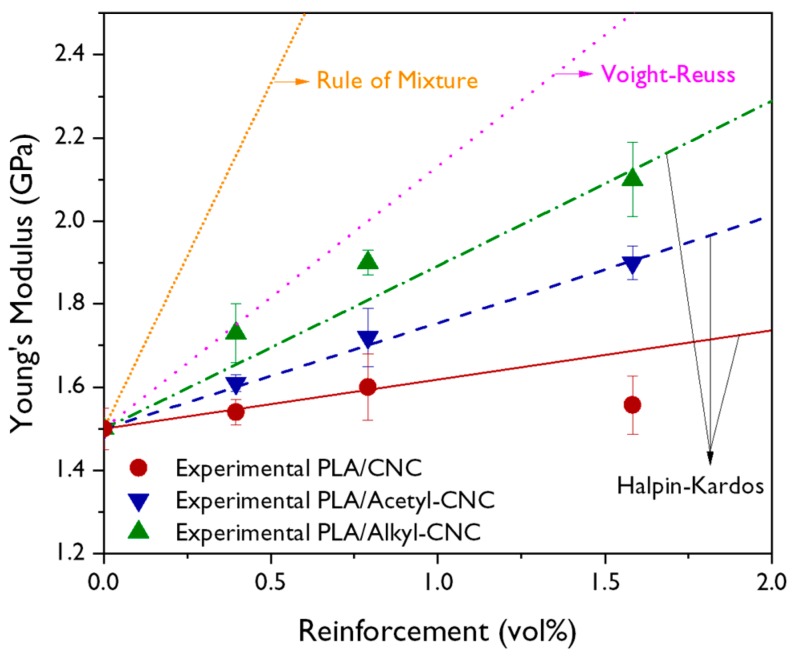
Theoretically predicted values and the experimental results for the Young’s modulus of the PLA nanocomposites.

The experimental data and theoretically predicted values for the modulus of PLA nanocomposites are compared in Figure 7. From the results, the Young’s modulus of PLA nanocomposites was found to be in good agreement with the Halpin-Kardos model, indicating that the applied stress was effectively transferred to individual CNCs, which have excellent mechanical properties. At lower alkyl-CNC content, the PLA nanocomposites exhibited higher values than values predicted from the Halpin-Kardos model. This implied that the strong interfacial adhesion achieved by alkylation of CNCs was more effective in improving the mechanical properties of the PLA nanocomposites than acetylation of CNCs was. Therefore, the addition of the alkyl-CNC into the PLA matrix resulted in the enhancement of interfacial adhesion as well as good dispersion, thereby improving the overall mechanical properties of the PLA nanocomposites.

#### 3.2.5. Barrier Properties

**Figure 8:**
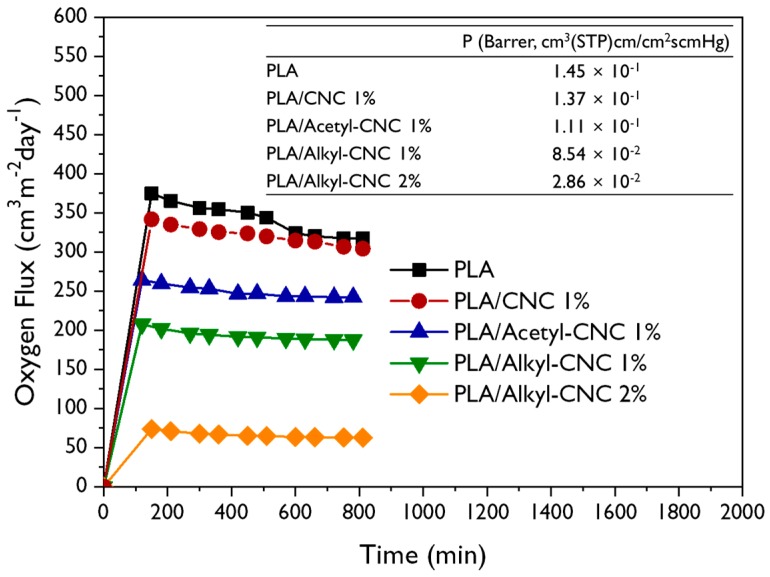
Oxygen flux rate and oxygen permeability coefficient of the neat PLA and PLA nanocomposites.

The barrier properties of the neat PLA and the PLA nanocomposites were evaluated using oxygen permeability analysis. Results are shown in Figure 8. The oxygen permeability coefficient (*P*), in the Barrer unit (1 Barrer = 10^−10^ cm^2^/s cmHg) is calculated by following equation [31].

(9) P=OTR×t/Δp

where *OTR* is the equilibrium oxygen transmission rate, *t* is the film thickness, and Δ*p* is the pressure gradient. The incorporation of modified CNCs decreased the *p* value, compared to *P* of the neat PLA, indicating enhancement of the barrier properties. In particular, the oxygen permeability of the PLA/alkyl-CNC nanocomposites was reduced to a greater extent than that of the PLA/acetyl-CNC nanocomposites due to the strong interfacial interaction between PLA and alkyl-CNC. Furthermore, the PLA/alkyl-CNC nanocomposites had a higher transmittance value in the visible region than the PLA/acetyl-CNC nanocomposites (Supplementary Figure S4). These results suggest that the addition of alkyl-CNC to the PLA matrix has a positive effect, in terms of suitability of utilizing the PLA nanocomposites for food packaging materials.

## 4. Conclusions

Facile surface alkylation of CNCs with an alkyl bromide via a simple S_N_2 reaction was successfully performed. The reinforcing effect in PLA nanocomposites was investigated, and compared with typical acetylation of CNCs. The surface modifications of CNCs resulted in remarkable enhancement of hydrophobicity, dispersion state, and interfacial compatibility with the PLA matrix. The activation energy for thermal degradation of the PLA nanocomposites indicated that the incorporation of a very small quantity of the modified CNCs into the PLA matrix improved the thermal stability of the PLA and that this effect was more pronounced in the case of the PLA/alkyl-CNC nanocomposites. Furthermore, a small amount of alkyl-CNC led to a significant improvement in the mechanical and gas barrier properties of the PLA nanocomposites because of uniform dispersion and strong interfacial adhesion. The moduli of the PLA nanocomposites were simulated using theoretical models. The Halpin–Kardos model was more suitable for PLA nanocomposites than the Voight-Reuss model. The reinforcing potential of modified CNCs in the PLA nanocomposites was confirmed. This efficient surface modification of CNCs is expected to be applicable to the development of lightweight, high-performance, and eco-friendly nanocomposite materials. In addition, according to the result of permeability analysis, the PLA/alkyl-CNC nanocomposites can be applied as food packaging applications.

## Figures and Tables

**Table 1:** The basic characteristics of surface modified CNC, identified by FT-IR and ^13^C NMR.

	Chemical Structure	Characterization
**FT-IR analysis**	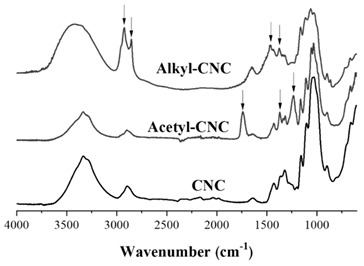	3350 cm^−1^: O-H band 1740 cm^−1^: C = O stretching 1370 cm^−1^: C-CH_3_ in-plane bending 1230 cm^−1^: C-O stretching 2853 and 2926 cm^−1^: C-H stretching
**Solid state ^13^C NMR analysis**	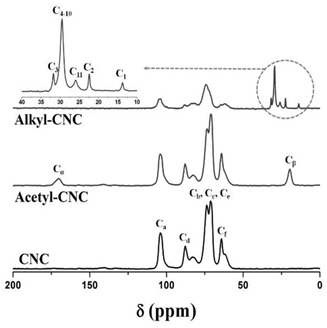	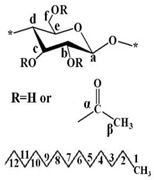

**Table 2:** Contact angle, *γ_S_^d^* (dispersion component), *γ_S_^p^* (polar component), and *γ_S_* (total surface energy) values of CNCs and modified CNCs.

	Water	Methylene Iodide	*γ_S_^d^* (mJ/m^2^)	*γ_S_^p^* (mJ/m^2^)	*γ_S_* (mJ/m^2^)
**CNC**	≤5°	20.1 ± 0.8°	32.2	41.4	73.6
**Acetyl-CNC**	78.3 ± 1.0°	35.3 ± 0.6°	38.1	4.14	42.2
**Alkyl-CNC**	84.9 ± 1.2°	33.8 ± 0.9°	40.8	1.73	42.5

**Table 3:** Thermal degradation parameters and activation energy of the neat PLA and PLA nanocomposites.

Materials	*T_5_*^a^ (°C)	*T_dm_* (°C)	*W_R_*^b^ (%)	Horowitz-Metzger	
				*E_a_* (kJ)	*r^2^*
**PLA**	310.5	357.3	0.7	211.7	0.99
**PLA/CNC 1%**	294.2	353.7	1.1	162.1	0.99
**PLA/Acetyl-CNC 1%**	328.1	365.8	1.2	282.6	0.99
**PLA/Alkyl-CNC 1%**	319.6	361.1	1.5	241.1	0.99

^a^ Thermal degradation temperature at 5% weight loss; ^b^ Residual yield in the TGA thermogram at 500 °C.

## Supplementary Materials

The following are available online at https://www.mdpi.com/2073-4360/12/1/178/s1.
