# Supplementary material for: Surface Alkylation of Cellulose Nanocrystals to Enhance Their Compatibility with Polylactide
Source: Polymers (Basel). 2020 Jan 9;12(1):178. doi: 10.3390/polym12010178 (PMC7022834; doi:10.3390/polym12010178)
Supplement: Supplementary file 1 [file polymers-12-00178-s001.pdf]

SUPPORTING INFORMATION

Surface Alkylation of Cellulose Nanocrystals to Enhance their Compatibility with Polylactide

Joo Hyung Lee[1, †], Sang Ho Park[2, †], and Seong Hun Kim[1, *]

[1]*Department of Organic and Nano Engineering,*

College of Engineering, Hanyang University, Seoul, Korea

[2]*LG Hausys R&D Center, Seoul, Korea*

*Corresponding Author: Prof. Seong Hun Kim, Ph.D. (E-mail: kimsh@hanyang.ac.kr)

†Both authors contributed equally to this work.

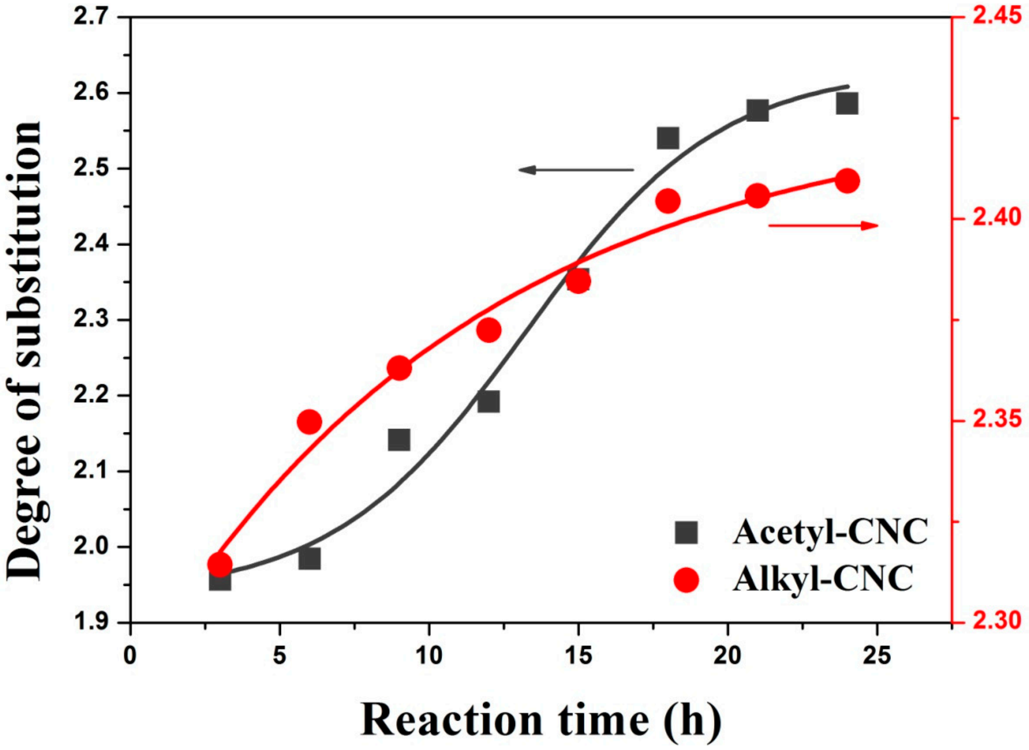

Figure S1. Degree of substitution of modified CNCs as a function of reaction time.

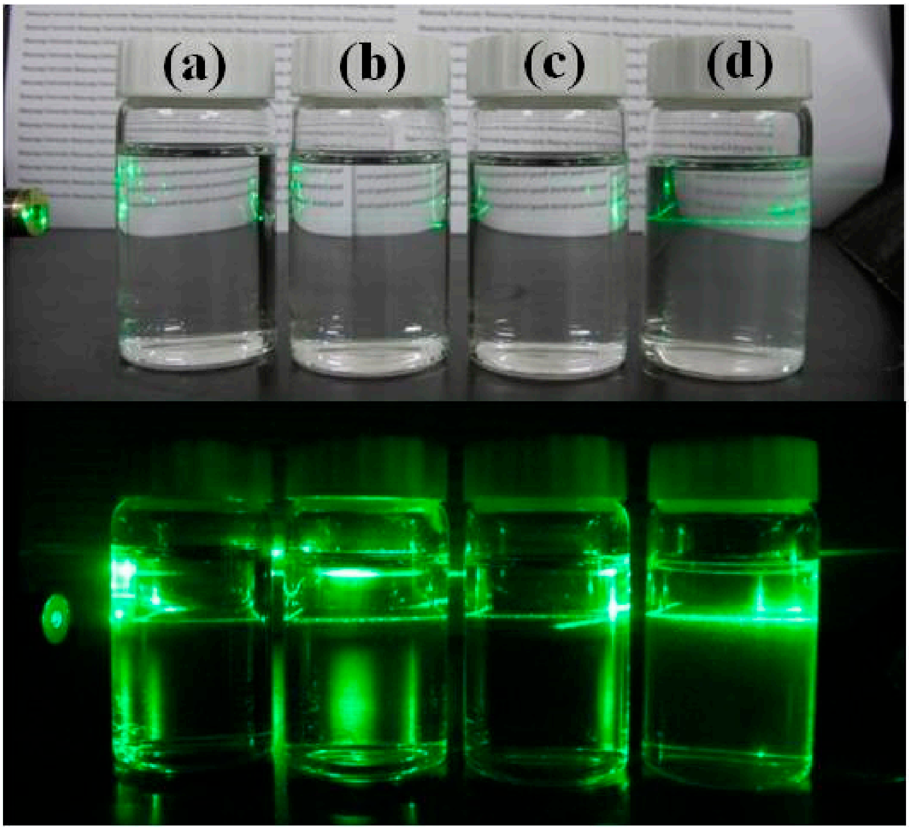

Figure S2. (a) Chloroform and dispersion states of (b) acetyl-CNC, (c) alkyl-CNC, and (d) CNC in chloroform.

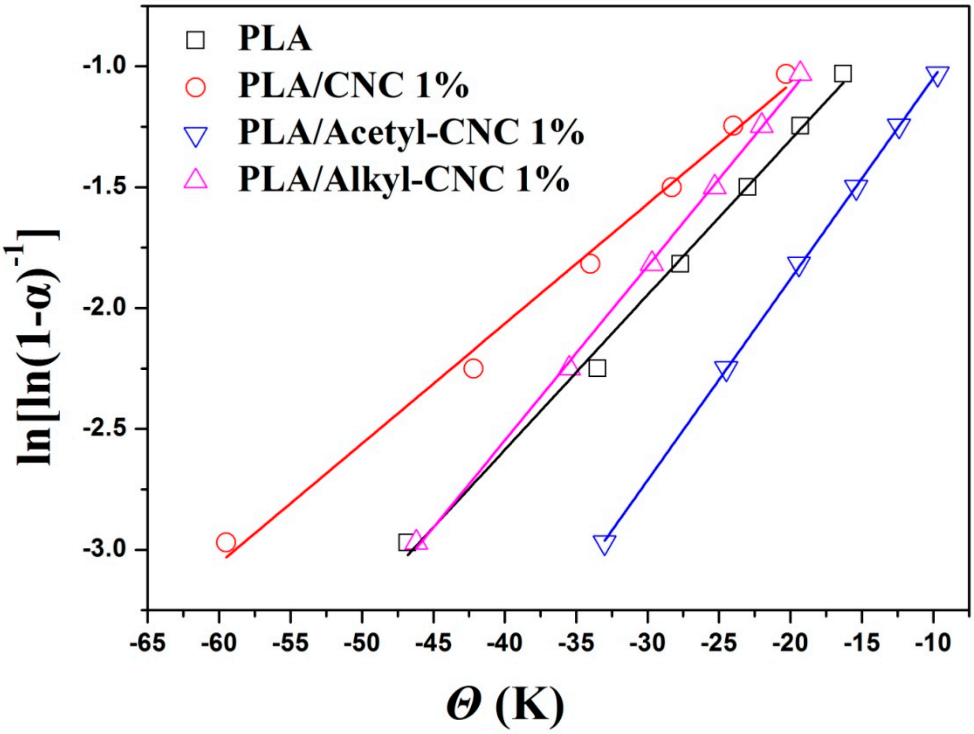

Figure S3. Horowitz-Metzger plots for obtaining E_a of the neat PLA and PLA nanocomposites.

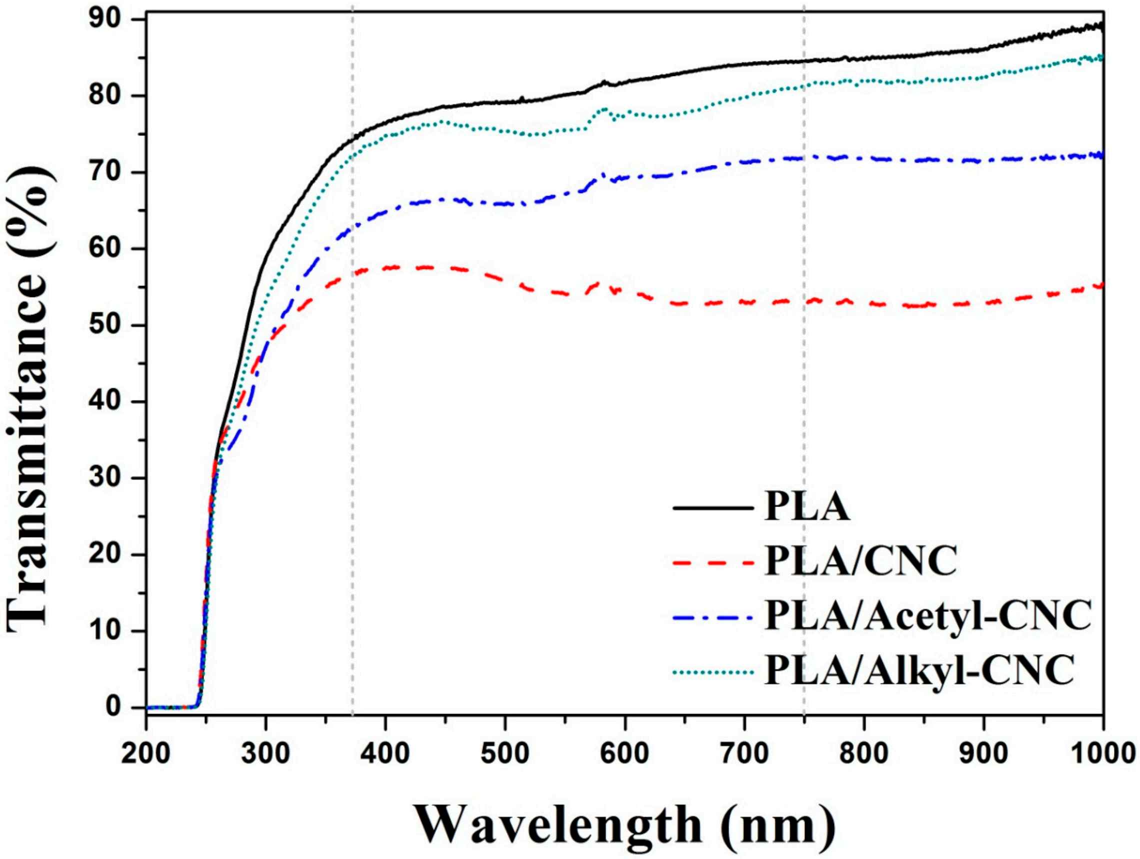

Figure S4. Transmittance curves for the neat PLA and PLA nanocomposites reinforced with 1 wt% of reinforcements.
